# State-of-the-Art Imaging in Human Chordoma of the Skull Base

**DOI:** 10.1007/s40134-018-0275-7

**Published:** 2018-04-03

**Authors:** Rene G. C. Santegoeds, Yasin Temel, Jan C. Beckervordersandforth, Jacobus J. Van Overbeeke, Christianne M. Hoeberigs

**Affiliations:** 1 Department of Radiology, Maastricht University Medical Center, P. Debyelaan 25, 6229 HX Maastricht, The Netherlands; 2 Department of Neurosurgery, Maastricht University Medical Center, Maastricht, The Netherlands; 3 Department of Pathology, Maastricht University Medical Center, Maastricht, The Netherlands

**Keywords:** Chordoma, Benign notochordal cell tumour (BNCT), Chondrosarcoma, Computed tomography (CT), Magnetic resonance imaging (MRI), Positron emission tomography (PET), Diffusion-weighted imaging (DWI)

## Abstract

**Purpose of Review:**

Chordoma are rare tumours of the axial skeleton which occur most often at the base of the skull and in the sacrum. Although chordoma are generally slow-growing lesions, the recurrence rate is high and the location makes it often difficult to treat. Both computed tomography (CT) and magnetic resonance imaging (MRI) are crucial in the initial diagnosis, treatment planning and post-treatment follow-up.

**Recent Findings:**

Basic MRI and CT characteristics of chordoma were described in the late 1980s and early 1990s. Since then, imaging techniques have evolved with increased resolution and new molecular imaging tools are rapidly evolving. New imaging tools have been developed not only to study anatomy, but also physiologic changes and characterization of tissue and assessment of tumour biology. Recent studies show the uptake of multiple PET tracers in chordoma, which may become an important aspect in the diagnosis, follow-up and personalized therapy.

**Summary:**

This review gives an overview of skull base chordoma histopathology, classic imaging characteristics, radiomics and state-of-the-art imaging techniques that are now emerging in diagnosis, treatment planning and disease monitoring of skull base chordoma.

## Introduction

Chordoma are rare tumours of the axial skeleton that occur most at the base of the skull and in the sacrum. Chordoma are thought to arise from remnants of the foetal notochord, that remain in the axial skeleton throughout life and may undergo malignant transformation into chordoma at any age [1, 2]. The incidence is estimated around 0.08 per 100,000 per year [3, 4], and chordoma account for around 6% of primary bone tumours [5]. The localization is distributed around 1/3rd skull base, 1/3rd spine and 1/3rd sacrum [3]. Although the overall 10y survival rate is around 55% [6], the 5- and 10-year recurrence rates are reported up to, respectively, 53 and 88% [7]. The most accepted therapy is resection as complete as possible. This is challenging at the level of the skull base due to surrounding critical structures including the optic system, carotid artery and brain stem. After the surgery, radiation with particle therapy has become the golden standard, i.e. proton beam or carbon-ion therapy [8]. Chordoma are resistant to conventional chemotherapy [9]. Therefore, adequate diagnosis and preoperative planning are crucial for maximal resection rate. This article describes the radiologic characteristics of chordoma and differential diagnosis and gives an overview of evolving imaging studies and future perspectives in radiology of this tumour entity. Understanding the histopathologic features and molecular markers is crucial for interpretation of imaging characteristics, especially in tumours with heterogeneous content like chordoma. Therefore, this review starts with a brief overview of the histopathology of these tumours.

## Histology and Immunohistochemistry

Macroscopically, chordoma appear as a white–grey soft, lobulated, gelatinous tumour with dens fibrous trabeculae [10–12]. Mucoid substance, necrotic areas, recent and old haemorrhages are found in the tumour, as well as calcification and sequestration of bone fragments [13]. The soft tissue is frequently surrounded by an incomplete pseudocapsule, with pressured surrounding tissue mimicking a true capsule [11].

There have been three different histological variants of chordoma described: classical (conventional), chondroid and dedifferentiated [14]. Dedifferentiated chordoma comprise < 4% of all chordoma subtypes [15], but have a much poorer prognosis [15–18]. The poorly differentiated chordoma are a particularly aggressive tumour with a predilection for the paediatric population [19]. Poorly differentiated or dedifferentiated chordoma may develop de novo, or transform as recurrent chordoma after surgery or radiotherapy [20, 21]. Although a study by Heffelfinger et al. [22] suggested that the chondroid variant has a better prognosis than classical chordoma, other studies [23–28] contradicted these results. Rosenberg et al. [27] redefined the histopathologic characteristics of the chondroid chordoma and concluded that part of the chondroid chordoma that were studied by Heffelfinger et al. [22] were actually low-grade chondrosarcoma. This makes a vital difference since chondrosarcoma tend to have a better prognosis than chordoma [29, 30].

All chordoma subtypes display a heterogeneous cytology. The predominant cell types are large cells which contain prominent solitary or multiple vacuoles, also called physaliphorous cells. These vacuoles are rich in mucopolysaccharides [22, 31, 32]. The smaller cells are non-vacuolated and referred to as stellate cells. The physaliphorous cells are arranged in sheets, cords or float singly within the abundant myxoid stroma and may be separated by fibrous bands [33, 34]. As seen macroscopically, chordoma are very heterogenous tumours with regional differences varying from areas of necrosis, to areas of mucoid degeneration, cartilage and connective tissue. Final diagnosis is made with immunohistochemistry. Chordoma show a unique immunohistochemical pattern with positive staining with antibodies against S100 protein, vimentin, low molecular cytokeratins and epithelial membrane antigen [22, 26]. The chondroid variant of chordoma is histologically similar to chondrosarcoma, and also share an immunoreactivity with antibodies against S100, which makes it challenging in differentiating between the two tumours. More recent studies show that brachyury, a T-box transcription factor, is a novel discriminating marker for chordoma [35, 36]. However, the role of brachyury in the pathogenesis of chordoma is still not completely understood.

## CT and MR Imaging Characteristics

**Fig. 1:**
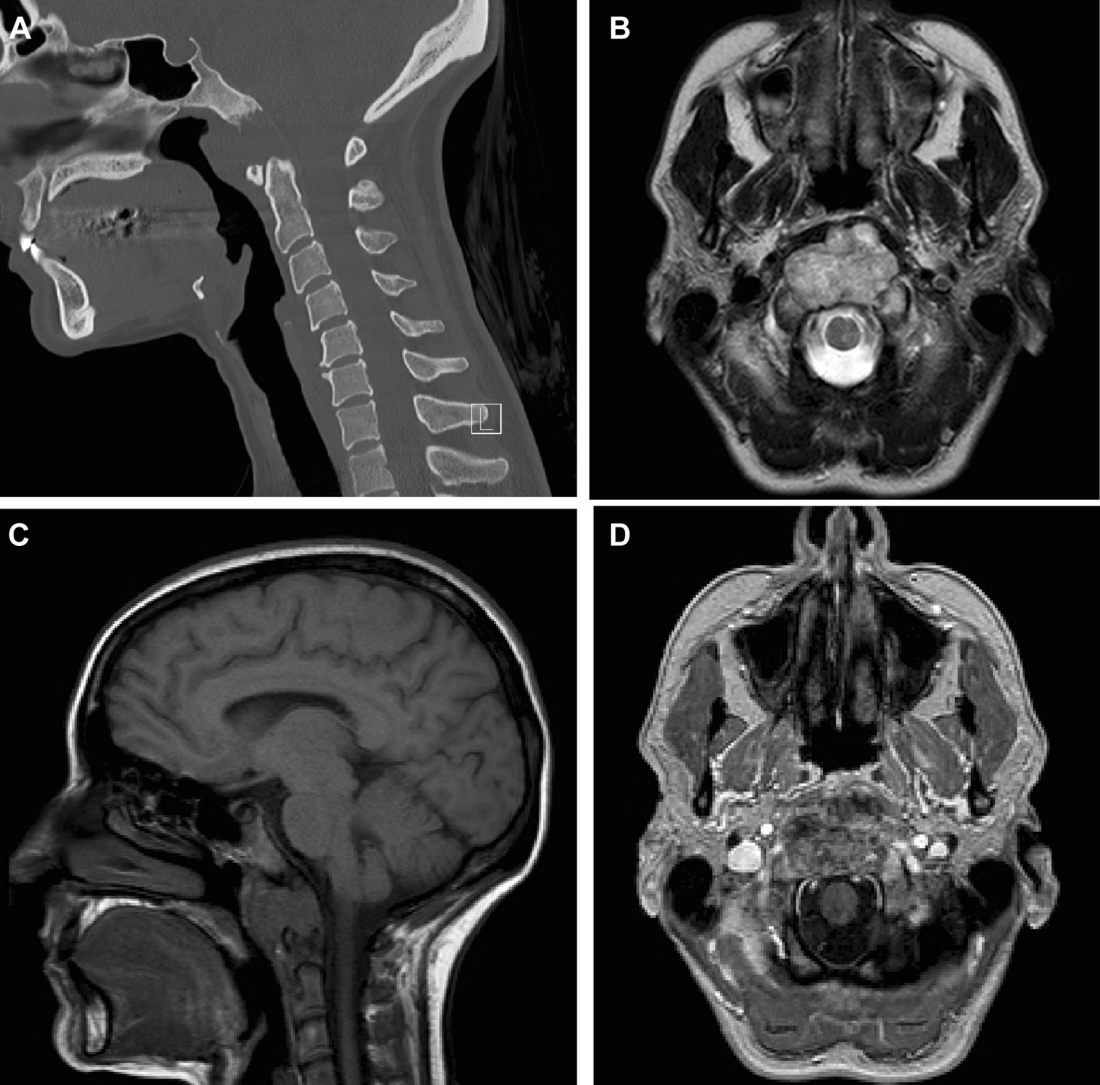
Chordoma. Imaging of a chordoma at the craniocervical junction. Computed tomography (**a**) shows lysis of the lower clivus. Axial T2-weighted imaging (**b**) shows a heterogenous, mostly hyperintense mass at the midline of the lower clivus. There is anterior extension to the oropharynx. The mass is hypointense on T1-weighted imaging (**c**) with heterogenous contrast enhancement (**d**). Pathologic examination confirmed the diagnosis of chordoma

Chordoma present as osteolytic, destructive lesions with associated cortical destruction and soft tissue extension. Chordoma of the skull base are generally located in the upper half of the clivus and often extends to the lower half of the clivus, posterior clinoid process, cavernous sinus and occipital condyle [37]. Sometimes chordoma may also arise at the craniovertebral junction and involve the atlas and axis (see Fig. 1). The bulk of the tumour is usually located in the midline of the patient. [38] Extraosseous extension of the tumour can occur in all directions, from the nasal cavity to indenting of the pons, also called thumb-sign [39]. Chordoma are slow-growing lesions [1]. Although case reports have described intradural location, chordoma are usually extradural lesions [40, 41].

On non-contrast CT, chordoma typically appear as well-circumscribed, hypoattenuating, heterogeneous lesion with extensive lytic bone destruction [42, 43]. The bulk of the tumour is usually hyperattenuating relative to the adjacent neuronal axis [44]. It may be difficult to distinguish between intratumoral calcifications, which are characteristic of the chondroid variant of chordoma [14], and sequestered fragments of the destroyed clival bone. There may be some separated areas of low attenuation within the tumour, probably also related to mucinous content [45].

**Fig. 2:**
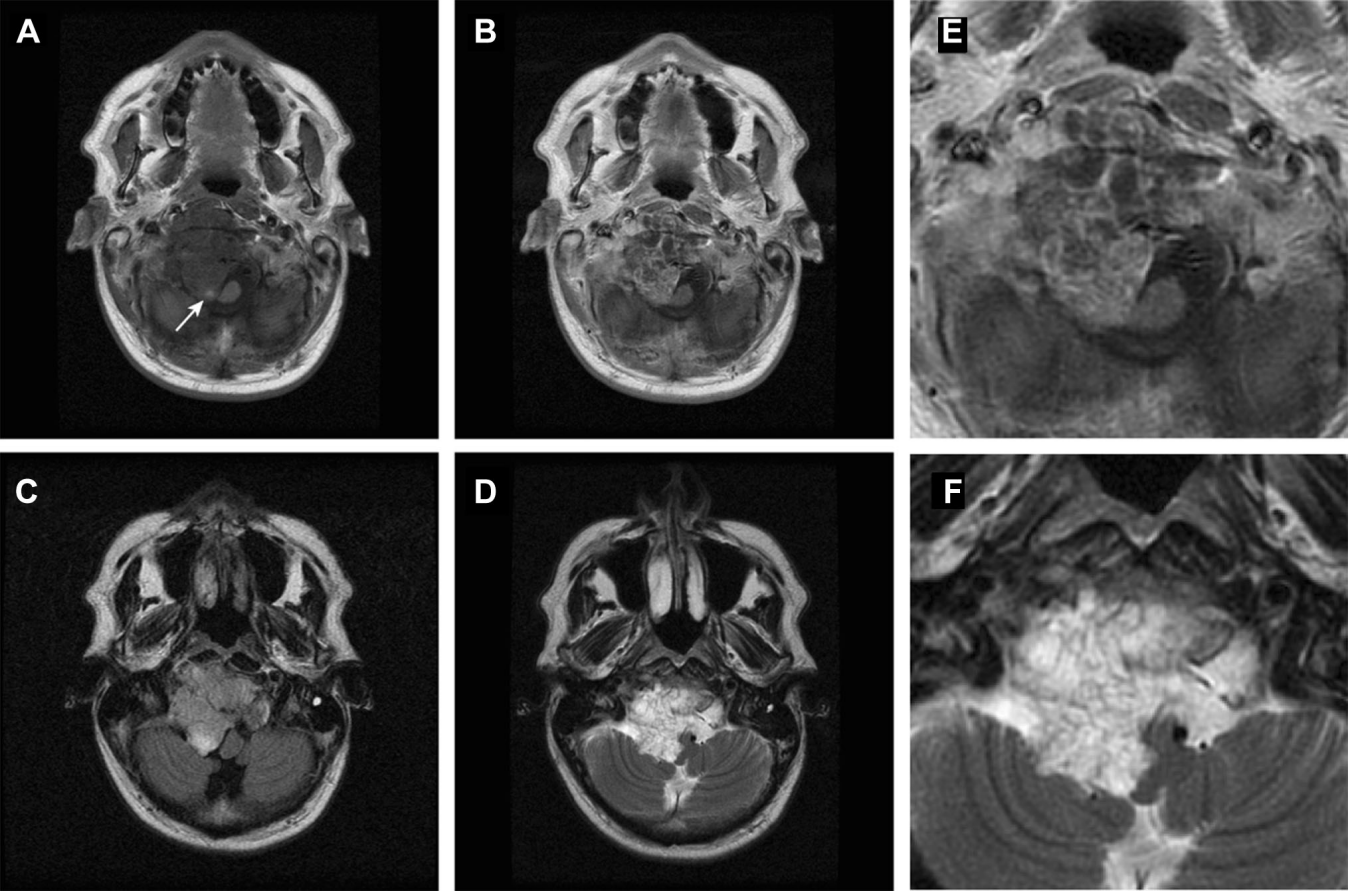
Chordoma. Magnetic resonance imaging of a skull base chordoma arising at the clivus with compression of the brain stem. Axial T1-weighted imaging (**a**) shows a mass with mostly hypointense signal, a hyperintense focus (arrow) and heterogeneous contrast uptake (**b**, **e**). Axial FLAIR (**c**) shows heterogenous tumour intensity. T2-weighted imaging (**c**, **f**) shows mostly hyperintense signal intensity with linear foci of hypointensity. Pathologic examination confirmed the diagnosis of chordoma

The MRI characteristics of chordoma with the standard MRI sequences have been well described as early as the late 1980s and early 1990s [46–50]. Since then, MRI has come a long way. New developments in acquisition techniques, MRI detectors and the use of higher magnetic fields are pushing MRI resolution to almost histological levels. Chordoma can have variable signal intensity on T1, classically generally low-to-intermediate signal intensity, with sometimes small foci of hyperintensity, correlated with mucus or haemorrhage [46]. Classic chordoma show high T2 signal intensity with heterogeneous hypointensity, which may also be associated with mucous, haemorrhage and also calcification. The presence of haemorrhagic foci or calcification can be confirmed with gradient echo images or susceptibility weighted imaging (SWI), showing susceptibility artefacts [46]. Low signal intensity septations may be seen, which may correlate with areas of necrosis or cartilage seen in histology. Poorly differentiated chordoma may show different imaging characteristics. Yeom et al. [51•] showed hypointensity on T2-weighted images in three poorly differentiated chordoma. However, there is a lack of studies in larger poorly differentiated chordoma cohorts. Chordoma typically show moderate to marked Gadolinium contrast enhancement with honeycomb appearance, with linear areas of non-enhancement [52]. This may also be explained with the areas of necrosis, connective tissue or cartilage in the tumour at histology. Fat suppression imaging with suppression of the fatty bone marrow of the clivus may be useful in the delineation of clival chordoma [44]. Figures 1 and 2 show typical imaging characteristics of chordoma. More advanced imaging techniques will be discussed later.

Metastases occur in 6–30% of all chordoma [53–56]. Common metastatic sites are lungs, bone, skin and liver [53, 55–57]. Chordoma metastases have similar imaging characteristics as the primary site. On CT images, chordoma metastases are generally low-density masses, as chordoma are typically osteolytic lesions. Similar to MRI of the primary tumour, chordoma metastasis show low-to-intermediate signal intensity on T1, high signal intensity on T2-weighted images and contrast enhancement in almost all cases [54]. Diffusion-weighted images show high signal intensity of the chordoma metastasis, most likely due to the gelatinous structure of chordoma [54]. The importance of diagnosing chordoma metastasis remains questionable as prognosis is more related to local disease at primary site than metastasis [53, 54].

## Role of Nuclear Medicine

The role of molecular imaging in diagnosis or treatment of chordoma is scarcely explored. Mirra et al. [58] described no abnormalities of chordoma on bone scans. To our knowledge, there are no studies of other PET tracers of skull base chordoma. Few studies have been performed in PET tracers of mobile spine and sacral chordoma. As skull base chordoma have similar molecular expression [35] as mobile spine and sacral chordoma, these tumours may be considered the same entity. Case reports show a heterogenous moderate pathological uptake of fluorodeoxyglucose (^18^F-FDG) uptake (SUVmax 4.5) within the primary tumour mass of a sacrococcygeal chordoma [59, 60]. These studies suggest that FDG-PET may aid in the diagnosis of chordoma metastasis. A recent study [61] showed ^18^F-FDG uptake in chordoma metastases, as well as ^68^GA-DOTA-TATE uptake in one chordoma with metastases. As stated earlier, the clinical relevance of diagnosing metastases is questionable as prognosis is more related to local disease control than metastasis [53, 54]. Nonetheless, ^18^F-FDG-PET is used in the follow-up of chemotherapy studies in chordoma, and may become an important aspect in future follow-up of chordoma patients [62].

Fluoromisonidazole positron emission tomography (FMISO-PET) is used in delineating areas of hypoxia in tumours [63]. Hypoxic areas in tumours are generally difficult to treat, as these areas are generally radioresistant and show increased resistance to cytotoxic chemotherapy [64]. The knowledge of hypoxic areas can be used in radiotherapy dose painting with boost volumes on these hypoxic areas. The feasibility of FMISO-PET-CT-guided radiotherapy with hypoxia-directed intensity modulated radiotherapy has been demonstrated in multiple tumours [63, 65, 66], including head and neck cancer [67]. Cheney et al. [68] showed that hypoxic areas could be identified with FMISO-PET-CT in 12 out of 20 chordoma of the mobile or sacrococcygeal spine. The benefits and risks of hypoxia-guided boost volumes in radiotherapy of chordoma still have to be investigated in the future.

## Differential Diagnosis

**Fig. 3:**
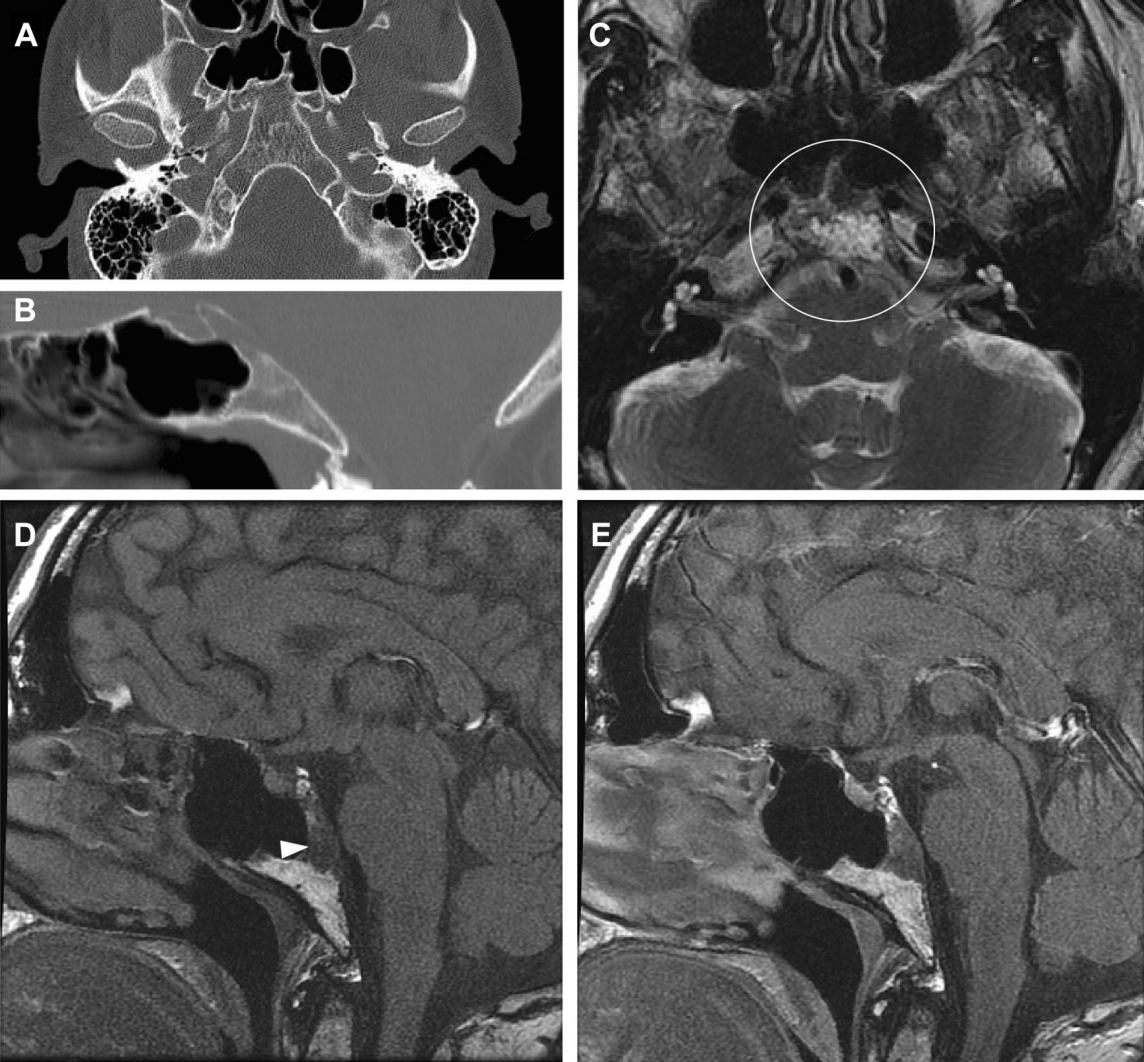
BNCT. Imaging of a well-defined intraosseous lesion in the base of the skull. CT imaging of the clivus in axial (**a**) and sagittal (**b**) plane shows normal bone trabeculation. There is no trabecular or cortical destruction. T2-weighted MRI imaging in axial plane (**c**) shows a hyperintense mass at the clivus, which is hypointense on T1-weighted imaging (arrow, **d**) and does not show contrast enhancement (**e**), as opposed to the physiological contrast enhancement of the pituitary gland. This tumour shows typical imaging characteristics of a benign notochordal cell tumour. However, there was no pathologic confirmation. On follow-up MRI after 1 year, the lesion was unchanged

As stated earlier, chordoma are assumed to arise from remnants of the foetal notochord. The notochord is a rod-shaped embryologic structure that develops in the third week of gestation, and serves as an molecular inducer for vertebral column formation [69]. As the vertebral column develops, the notochord degrades into clusters in what later becomes the nucleus pulposus [70]. Notochordal cells are normally found in the intervertebral disc in the postnatal period until the early adult life [71]. However, embryological studies of human foetuses have demonstrated ectopic notochordal remnants along the axial skeleton, and especially at the caudal and cranial ends [72]. It is thought that these notochordal remnants may proliferate into chordoma. Over the years, multiple benign tumours with notochordal cell origin have also been described, i.e. ecchordosis physaliphora [73], giant notochordal rest [74–76], giant notochordal hamartoma [58] and are now usually categorized in the comprehensive term ‘benign notochordal cell tumours’. These tumours are thought to be proliferated remnants of the notochord, with a benign character [58, 77]. It is debated whether a BNCT may undergo malignant transformation to chordoma [78–81]. In autopsy studies performed by Yamaguchi et al. [80], 20% of the cadavers showed foci of BNCT in the axial skeleton. It should be emphasized that > 50% of these foci were < 2 mm in size and are probably not seen in imaging. Benign notochordal cell tumours and chordoma differ in radiologic and histologic features. On computed tomography, BNCTs are usually occult or show slight sclerosis without bone destruction [82]. On MRI, BNCTs show low T1 signal intensity and intermediate-to-high signal intensity on T2-weighted images and are almost exclusively intraosseous without soft tissue extension or contrast enhancement [83, 84]. Figure 3 shows an intraosseous lesion with the imaging characteristics of a BNCT. Multiple studies suggest imaging follow-up when a lesion is suspect of BNCT [1, 82]. However, because of the huge difference in the prevalence of BNCT and incidence of chordoma (20% versus 0.08/100,000 per year), follow-up imaging may only be necessary if the lesion shows atypical BNCT imaging characteristics, like bone destruction. However, there are no long-term studies in follow-up of BNCT.

**Fig. 4:**
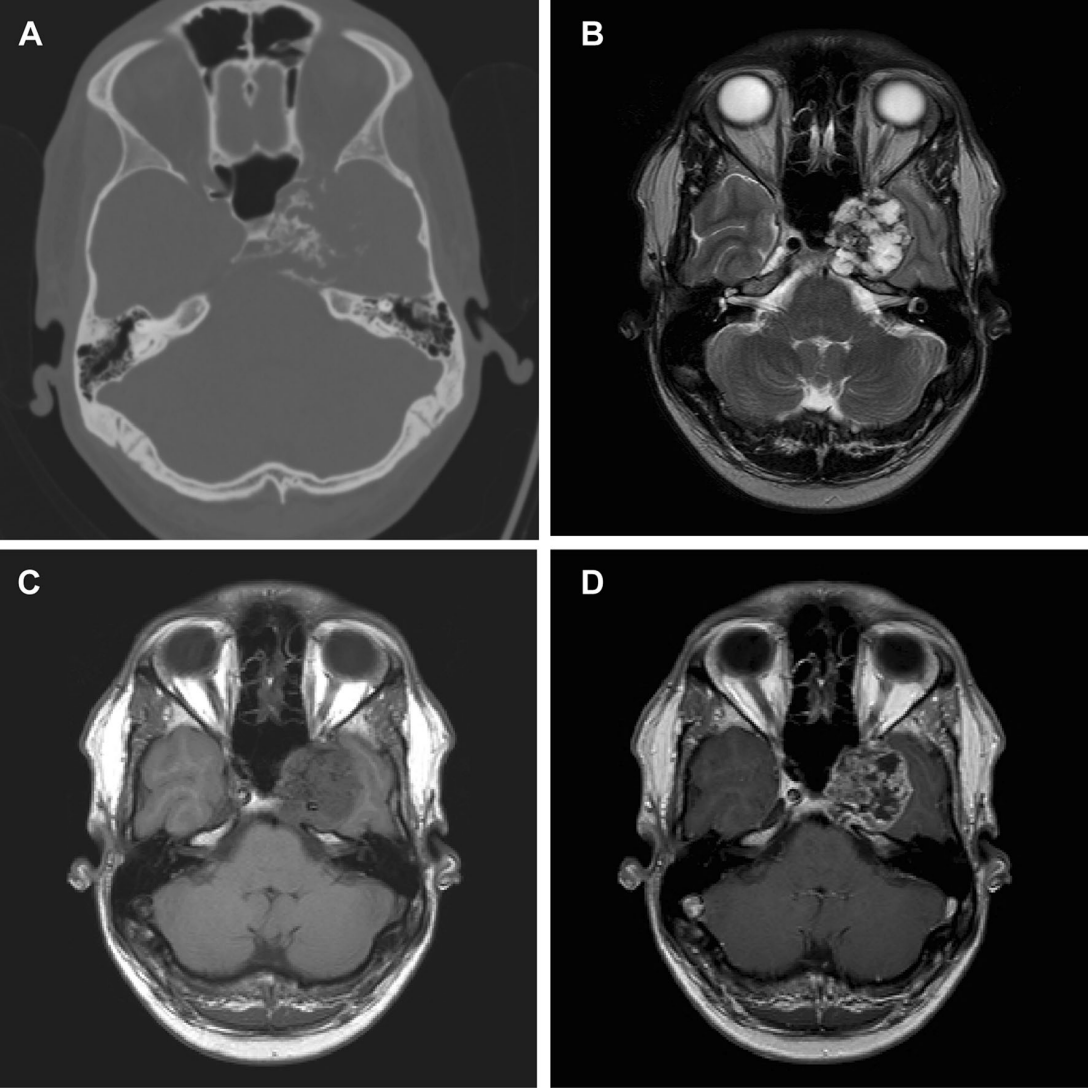
Chondrosarcoma. Imaging of skull base chondrosarcoma arising from the left lateral clivus with extension to the clinoid process, cavernous sinus and intracranial extension and compression on the left temporal lobe. Computed tomography (**a**) shows bone destruction with hyperdense fragments in the tumour mass, which corresponds with the typical ‘rings-and-arcs’ seen in chondroid tumours like chondrosarcoma. T2-weighted imaging (**b**) shows a heterogenous, mostly hyperintense mass. T1-weighted imaging (**c**) shows a hypointense mass with heterogenous enhancement (**d**). Pathologic examination of the tumour mass concluded chondrosarcoma WHO grade 1

Of all benign notochordal cell lesions, ecchordosis physaliphora (EP) are a subtype that was already described in the 19th century. EP are described as a congenital, benign, gelatinous tissue that is considered an ectopic notochordal remnant [85]. Ecchordosis physaliphora are found in around 2% of autopsy studies [73] and approximately 1.7% in MRI studies [85, 86]. Even though this lesion appears to be common, it is easily overlooked. Typically, EP are small, well-circumscribed, extra-axial, intradural lesions in the prepontine cistern, which have a predilection for the midline posterior clivus at the level of Dorello’s canal [85, 87, 88]. On MRI, EP show high signal intensity on T2-weighted images and low signal intensity on T1-weighted images, similar to chordoma, but do not show contrast enhancement [85, 86, 88–92]. Differentiating EP from intradural chordoma is essential, as EP are benign and usually asymptomatic and therefore do not need treatment [93].

Chondrosarcoma are the most difficult differential diagnosis of chordoma of the skull base, as these lesions have overlapping radiologic and histopathologic features [52]. Many previous studies have found no definite CT or MRI features that distinguish between chordoma and chondrosarcoma [38, 47, 52]. However, there are some features that favour one over the other. Chordoma typically originate in the clivus and are therefore generally located more centrally, whereas chondrosarcoma arise in the petroclival fissure and therefore occur more laterally. [38] Unfortunately, this is not a distinctive feature, as lateral skull base chordoma have also been described [94, 95]. On CT, chondrosarcoma may show a typical ‘ring-and-arc’ chondroid matrix mineralization [96]. Figure 4 shows typical imaging characteristics of a chondrosarcoma. Recent studies show that diffusion-weighted imaging may differentiate between chordoma and chondrosarcoma, with chondrosarcoma having a higher average apparent diffusion coefficient than chordoma [51•, 97, 98]. The use of DWI in chordoma imaging is further elaborated below in ‘prognostics and radiomics’.

Table 1 gives an overview of the typical imaging characteristics of chordoma, BNCT and chondrosarcoma.

**Table 1:** Imaging characteristics of chordoma and differential diagnosis

	T1	T2	Contrast enhancement	Bone destruction	Preferred location
Chordoma	Low to intermediate	High	Yes	Yes	Midline
Chondrosarcoma	Low to intermediate	High	Yes	Yes	Out of midline
BNCT	Low	High	No	No	Intraosseous, no soft tissue extension
EP	Low	High	No	No	Midline. Stalk with clivus. Extraosseous

## Post-treatment Evaluation

As chordoma show high recurrent rates [7], follow-up seems obvious. However, there is no evidence for any type of follow-up in chordoma. Stacchiotti et al. [99], on behalf of ‘the chordoma global consensus group’, proposed follow-up imaging of the primary tumour site with MRI during the first 4–5 years after diagnosis every 6 months. Thereafter, if no disease progression is observed, MRI should be done every year for at least 15 years. There was no consensus about follow-up of metastasis in this group. Guler et al. [100] studied the follow-up of chordoma using MRI with incorporating DWI and concluded that the detectability of residual chordoma tumour tissue on DWI is better than T2 or FLAIR sequences.

## Prognostics and Radiomics

As stated earlier, chordoma have a high recurrence rate, with up to 20% recurrence in the first year after diagnoses [7]. Unfortunately, prognostic factors in chordoma are largely unknown. Histopathologic studies have shown that poor differentiation type, high mitotic activity, prominent nucleoli, high Ki67 and high p53-LI significantly correlates with poor progression-free survival [101]. The presence of necrosis and apoptosis is related to poor overall survival [101]. Moreover, better overall survival is linked to the possibility of high resection rate and smaller tumour size [4]. Skull base chordoma show a better overall survival than sacral chordoma, most likely due to the smaller average sizes in skull base chordoma, as tumours in this location become symptomatic sooner and therefore detected at an earlier stage [4].

Little is known about prognostic imaging characteristics in chordoma. Large radiomics studies have not yet been performed. A few pilot studies have been conducted in chordoma imaging characteristics for prognostic and differential diagnostic purposes. A recent study performed by Tian et al. [102••] studied the tumour-to-pons signal intensity ratios of 156 patients with skull base chordoma and showed that a higher T2 signal ratio (tumour: pons) was an indicator of slow tumour progression, whereas a high enhanced T1 FLAIR ratio (tumour: pons) was a positive indicator of tumour progression. Low T2 signal intensity and high T1 contrast-enhanced signal intensity may be associated with abundant blood supply of the tumour. These ratios used in this study were also categorized in a gradient system which showed increased tumour progression with higher grade. Future prospective studies are needed to evaluate the efficacy of this grading system.

Diffusion-weighted imaging (DWI) is an unenhanced MRI technique based on the restriction of Brownian motion of extracellular water [103]. The apparent diffusion coefficient (ADC) is a quantitative measure of this movement. This difference can also aid in the characterization of tumorous tissue, as water molecule diffusion is reflective of tissue organizational features, i.e. cellularity, nuclear-to-cytoplasmic ratio and reduced extracellular matrix [104, 105]. It is believed that benign tumours have lower cellularity, whereas malignant tumours generally show high cellularity with high mitotic activity [106]. Studies show that quantitative DWI with ADC mapping can distinguish between benign and malignant musculoskeletal lesions of the skull [104, 105]. The myxoid stroma of chordomas, with nests of physaliphorous cells separated with fibrous strands may impede extracellular water motion [51•]. As stated earlier, DWI with quantitative ADC mapping may be used in differentiating chordoma from chondrosarcoma, with chondrosarcoma having a higher average ADC value than chordoma [51•,^97^, ^98^]. The mean value in a study of Yeom et al. [51•] was 2051 ± 261 × 10^−6^ mm^2^/s for chondrosarcoma, followed by 1474 ± 117 × 10^−6^ Ecchordosis physaliphora mm^2^/s for classic chordoma and 875 ± 100 × 10^−6^ mm^2^/s for poorly differentiated chordoma. This is in accordance with the premise that malignant tumours have higher cellularity and therefore lower ADC values. More research is needed to evaluate relationship of ADC values and prognosis in patients with chordoma.

A recent study by Lang et al. [107] used dynamic contrast-enhanced (DCE) MRI to differentiate chordoma from giant cell tumours of the skull base. Dynamic contrast enhancement is an MRI technique which calculates the perfusion of tissue [108]. A typical DCE kinetic pattern was described for chordoma which was significantly different from giant cell tumours. In this study, chordoma show typical quick enhancement without washout, as opposed to giant cell tumours, which show quick enhancement and washout. To our knowledge, there are no studies that evaluate the role of DCE-MRI in the differentiation of chordoma and other lesions of the skull base.

## Future Directions in Chordoma Imaging

The imaging characteristics of chordoma with CT and standard MRI sequences are well described in the late 1980s and early 1990s [46–50]. At that time, imaging was used for diagnostic and treatment planning purposes. The first molecular PET studies were performed halfway the 2000s. Molecular imaging data of chordoma with PET-CT are currently still limited but may become important in the future. The driving mechanisms behind chordoma are still largely unknown. Imaging may become more important in differentiating between slow-growing and fast-growing lesions, and therefore crucial in treatment planning. The presence of hypoxic areas in chordoma, which has been confirmed by FMISO-uptake [68], may be an indicator of poor prognosis. More research is needed to evaluate the role of hypoxia in the prognosis of chordoma and the benefit of hypoxia-adjusted radiotherapy.

Targeted radionuclide therapy (TRT) is a hot topic in cancer therapy nowadays. Coupling radionuclides to tumour-specific targeting agents, i.e. tumour-specific membrane receptors, delivers radiation to the cancer cells that express these membrane receptors. The discovery of ^68^GA-DOTA-TATE uptake in chordoma is exciting. ^68^GA-DOTA-TATE is a somatostatin receptor analogue, and ^68^GA-DOTA-TATE PET-CT plays an important role in the detection of primary tumour, metastases, staging, restaging and assessment of treatment response in patients with neuroendocrine tumours [109]. Studies show that ^68^GA-DOTA-TATE uptake in neuroendocrine tumours also predicts response to somatostatin receptor-mediated radionuclide therapy [110, 111]. More research is needed to evaluate the role of ^68^GA-DOTA-TATE in larger chordoma cohorts and the efficacy of treatment using somatostatin receptor-mediated radionuclides in chordoma.

To our knowledge, no studies have been performed to evaluate the use of PET-MR in chordoma. The high spatial resolution of MR combined with the molecular and functional information of PET may aid in diagnosis and surgery strategy planning. Further advancements in molecular imaging may aid in the differential diagnosis of skull base lesions. Radiolabelled molecular probes may be used to identify unique proteins in cancer cells [112], or even used as biomarkers. Molecular imaging by PET may function as a bridge between in vitro and in vivo biology of disease.

Imaging research is focussing more and more on the new emerging and rapidly evolving field of radiomics. Advanced imaging characteristics may become a key player in predicting cancer aggressiveness, or prediction of response or resistance to treatment [113]. This is already used in diagnosis, treatment planning and disease monitoring of brain tumours [114]. Radiomics is a field of imaging studies which uses quantitative imaging features, like signal intensity, size, shape and texture for information on tumour phenotype and microenvironment [115]. Assessing these imaging features and linking these to prognostic data may lead to calculation of individualized prognosis and tailor-made personalized treatment. Pilot studies in radiomics have been performed, but large studies are still missing. New state-of-the-art MRI tools, like DWI, spectroscopy, dynamic contrast-enhanced MRI and BOLD have barely been studied. Future studies are needed to evaluate the role of these techniques in diagnosis, treatment and follow-up of chordoma. Despite a large imaging arsenal, diagnosis and treatment response largely depend on semantic features based on conventional imaging. Advanced, hybrid techniques and quantitative measures are being implemented more and more and in the future can aid in increased personalized medicine.
